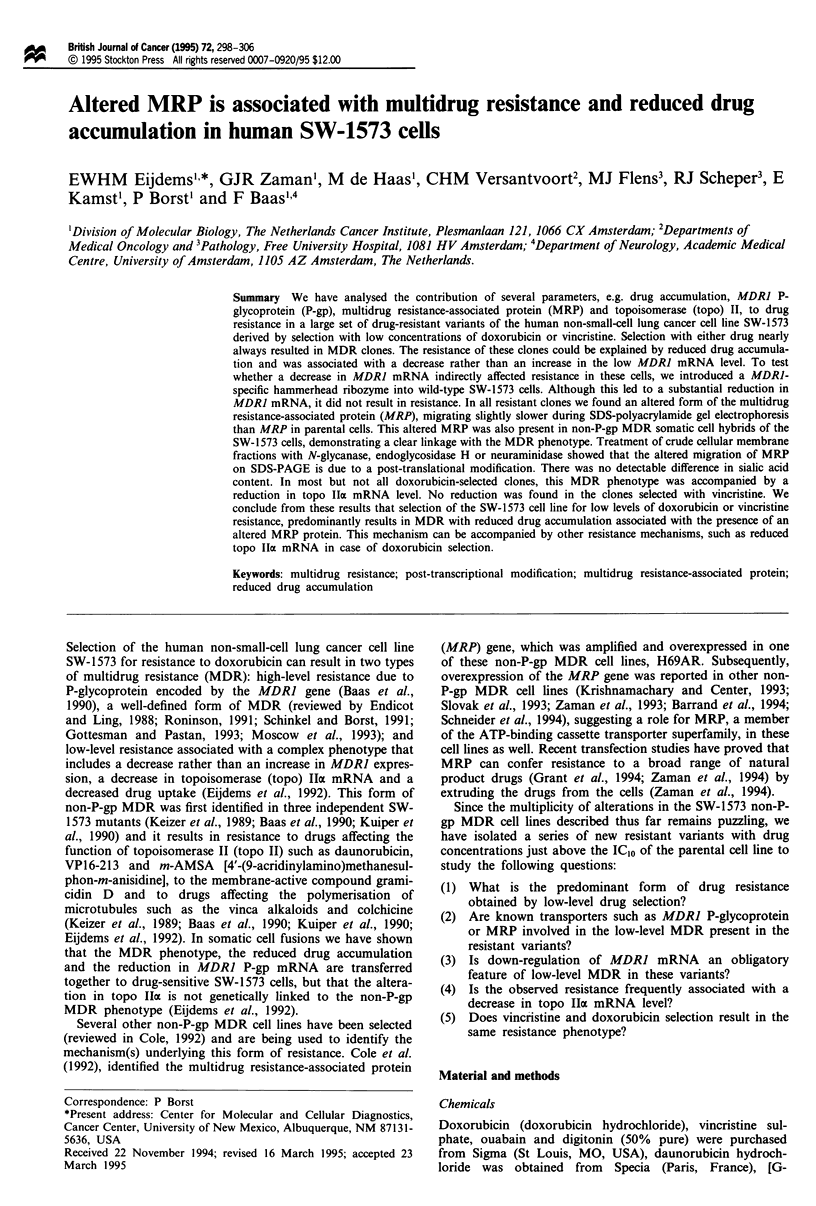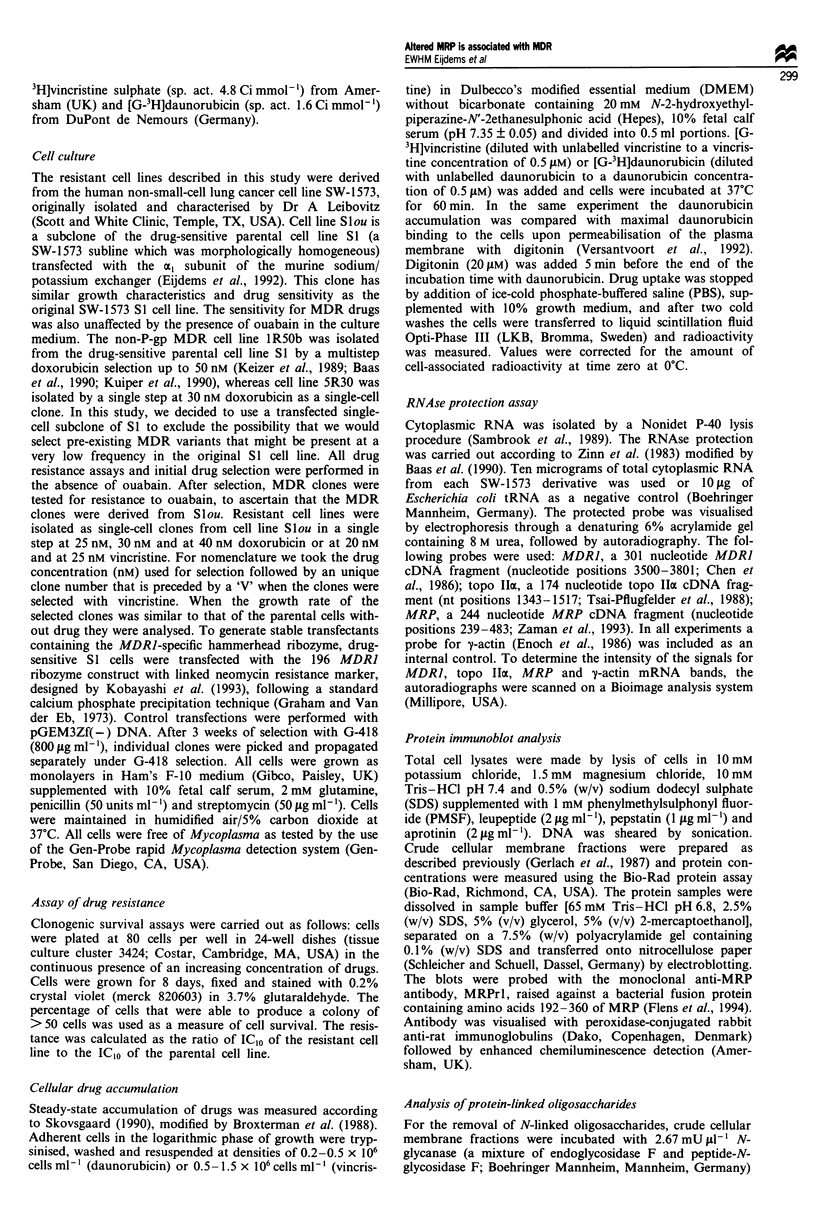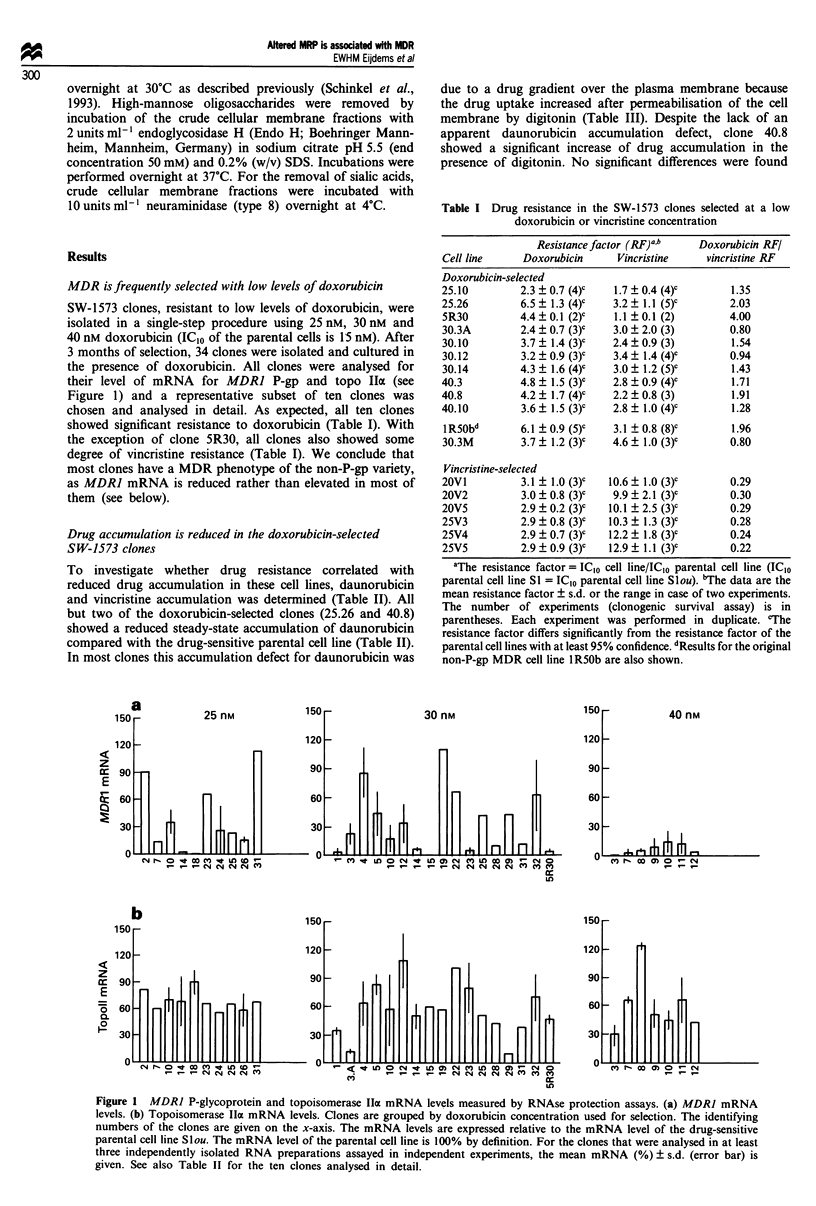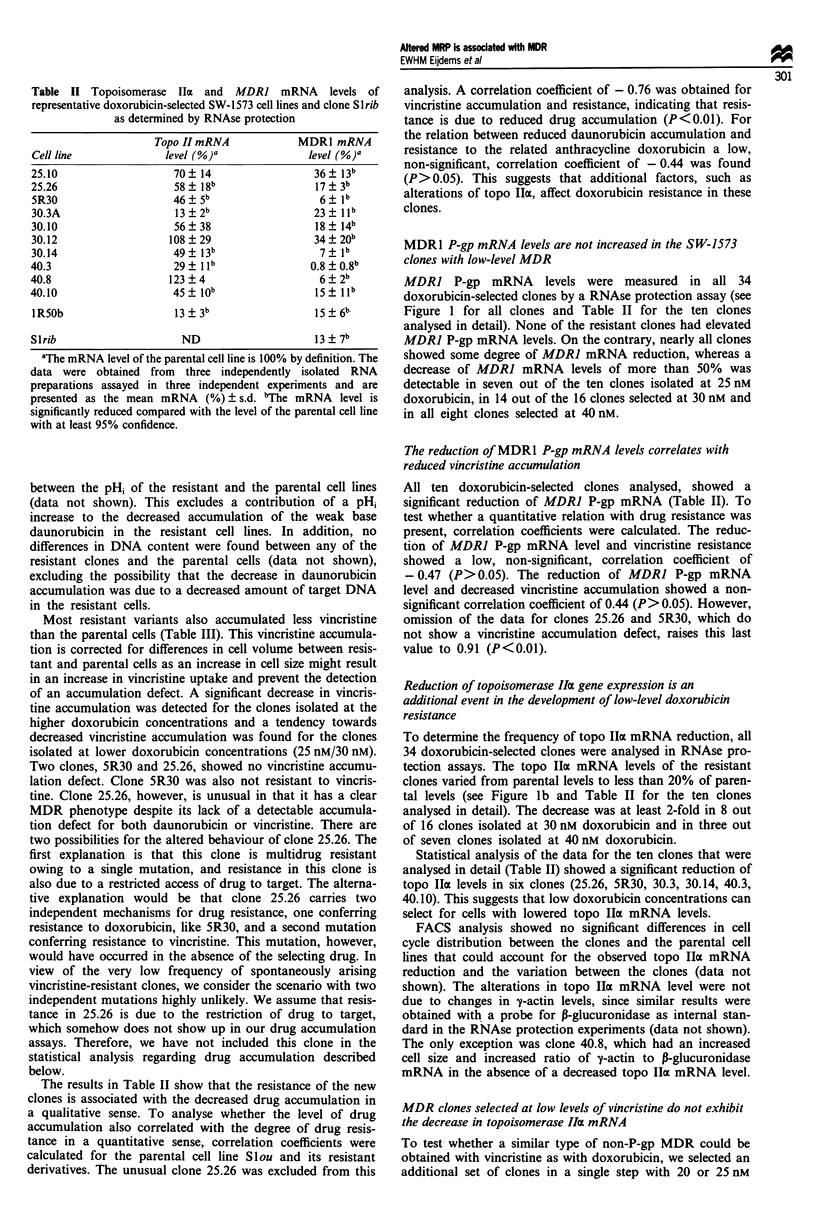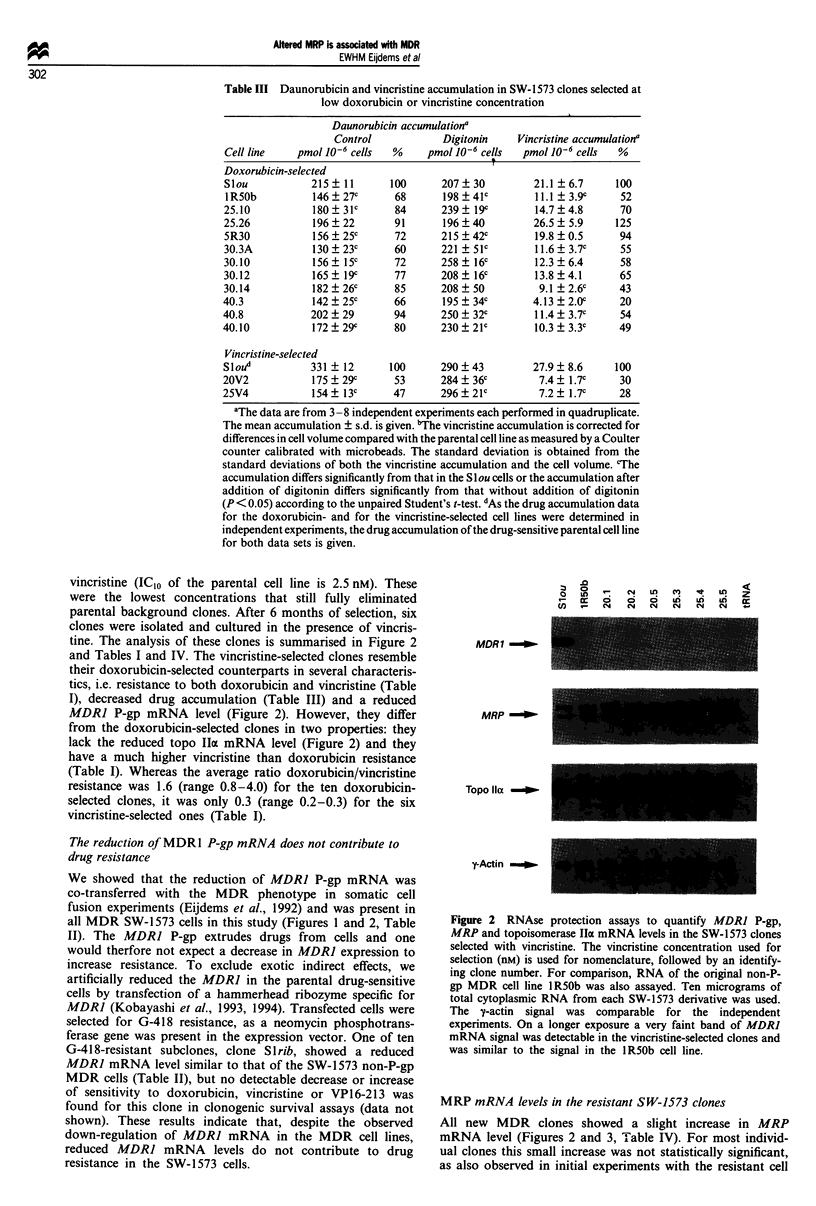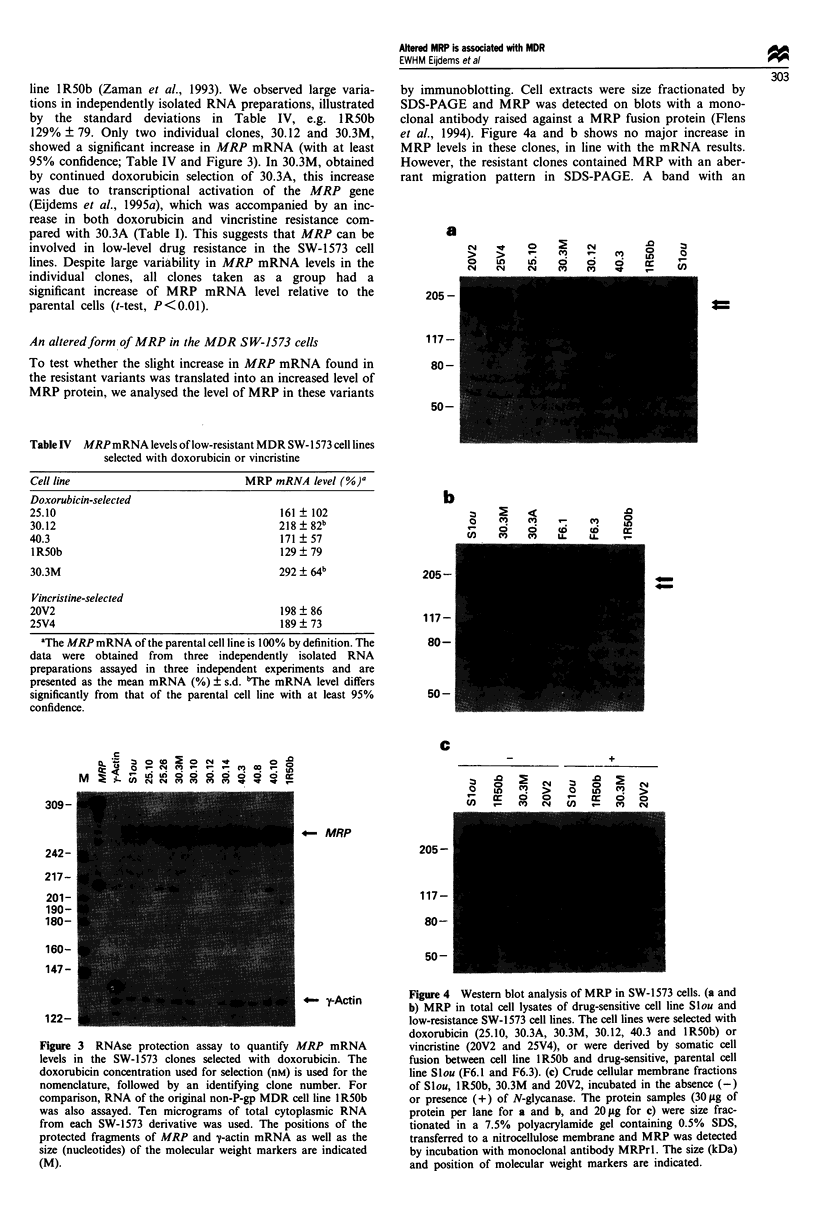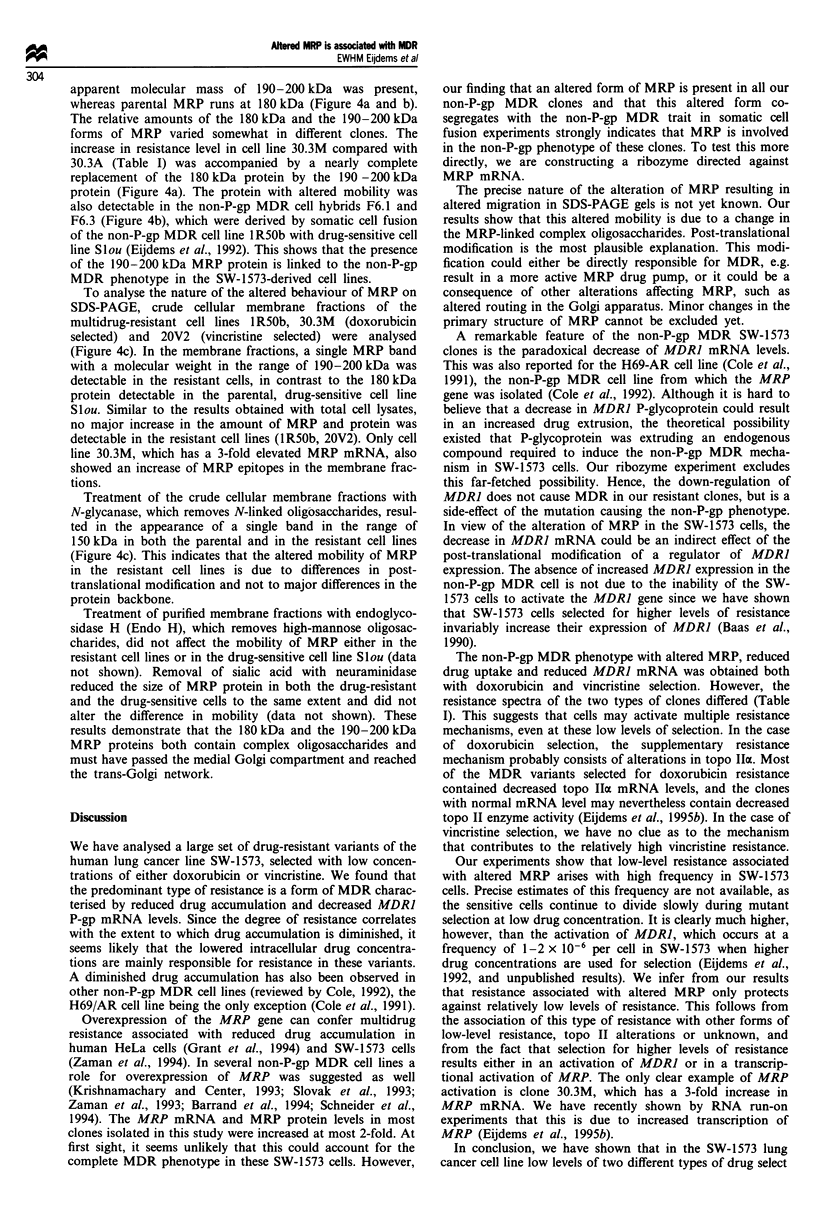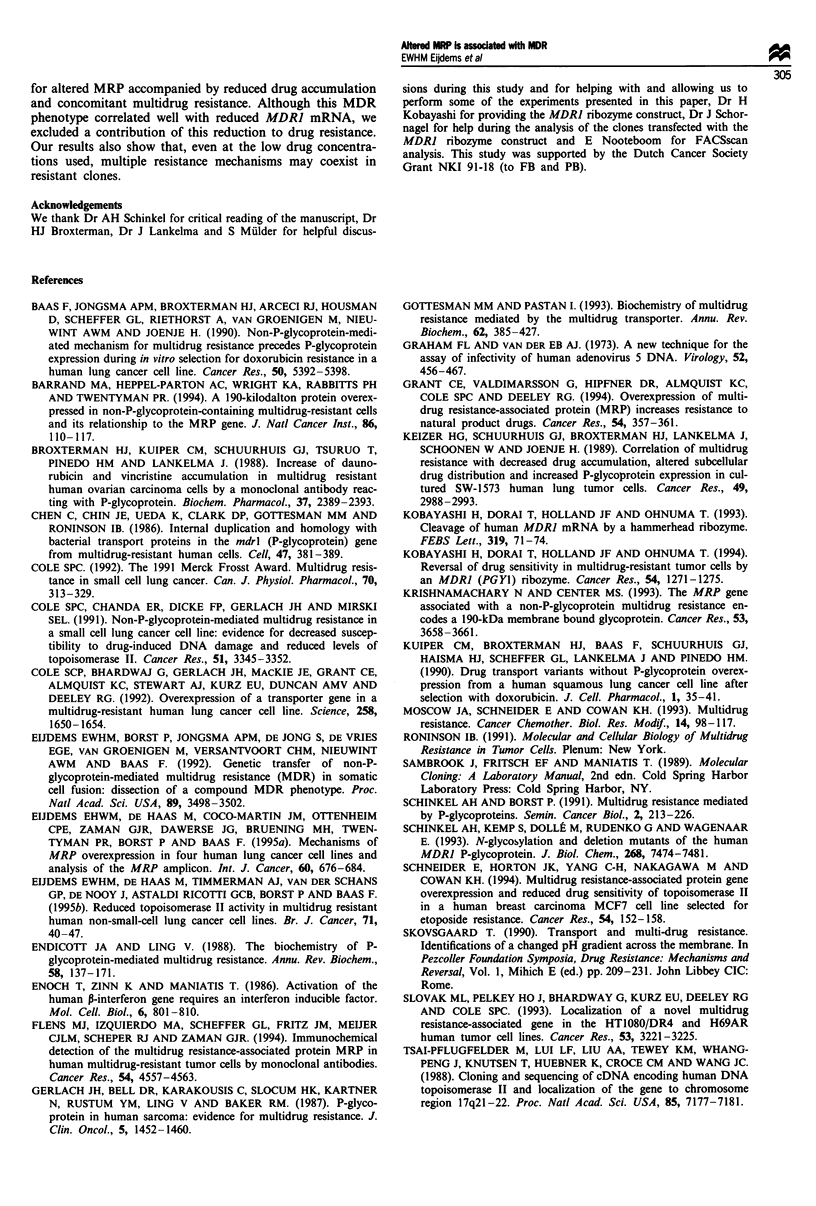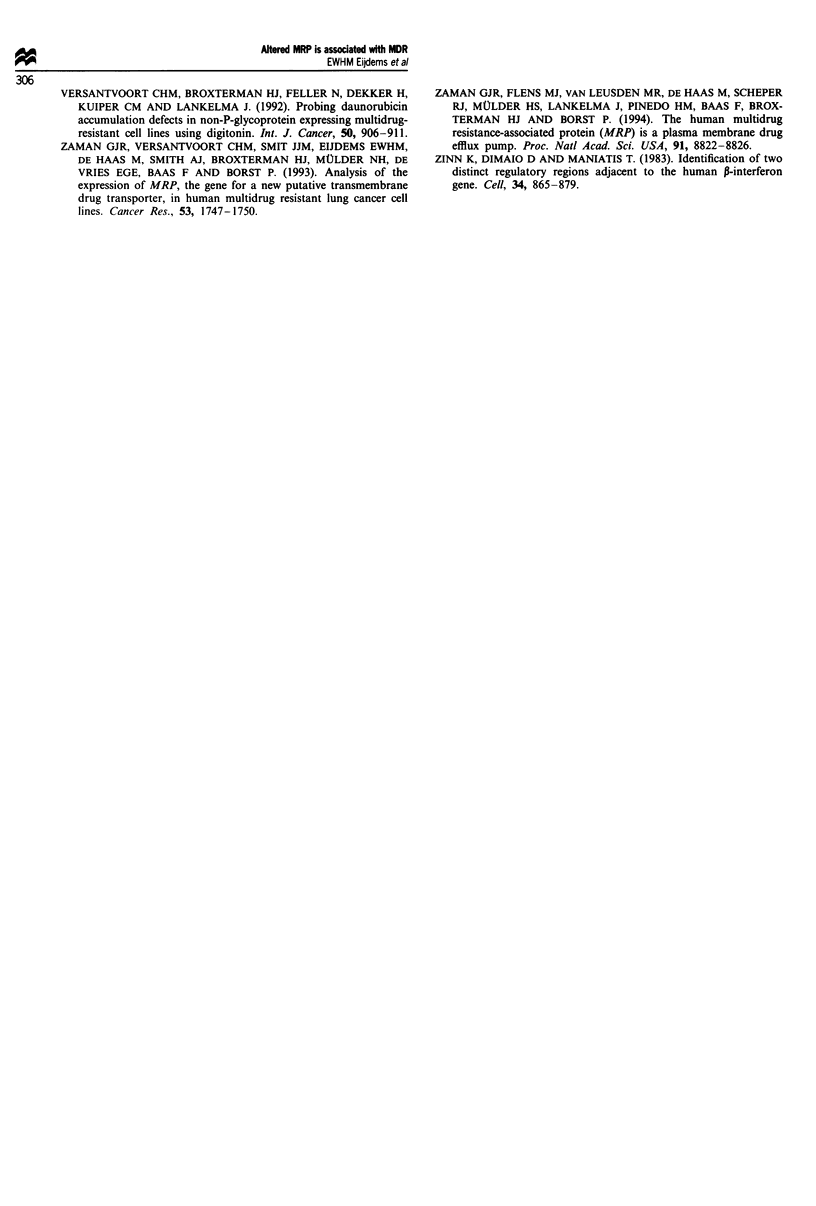# Altered MRP is associated with multidrug resistance and reduced drug accumulation in human SW-1573 cells.

**DOI:** 10.1038/bjc.1995.328

**Published:** 1995-08

**Authors:** E. W. Eijdems, G. J. Zaman, M. de Haas, C. H. Versantvoort, M. J. Flens, R. J. Scheper, E. Kamst, P. Borst, F. Baas

**Affiliations:** Division of Molecular Biology, The Netherlands Cancer Institute, Amsterdam.

## Abstract

**Images:**


British Journal of Cancer (1995) 72, 298–306
© 1995 Stockton Press All rights reserved 0007-0920/95 $12.00

### Altered MRP is associated with multidrug resistance and reduced drug accumulation in human SW-1573 cells

EWHM Eijdems[1,*], GJR Zaman[1], M de Haas[1], CHM Versantvoort[2], MJ Flens[3], RJ Scheper[3], E Kamst[1], P Borst[1] and F Baas[1,4]

[1]*Division of Molecular Biology, The Netherlands Cancer Institute, Plesmanlaan 121, 1066 CX Amsterdam;* [2]*Departments of Medical Oncology and* [3]*Pathology, Free University Hospital, 1081 HV Amsterdam;* [4]*Department of Neurology, Academic Medical Centre, University of Amsterdam, 1105 AZ Amsterdam, The Netherlands.*

**Summary** We have analysed the contribution of several parameters, e.g. drug accumulation, *MDR1* P-glycoprotein (P-gp), multidrug resistance-associated protein (MRP) and topoisomerase (topo) II, to drug resistance in a large set of drug-resistant variants of the human non-small-cell lung cancer cell line SW-1573 derived by selection with low concentrations of doxorubicin or vincristine. Selection with either drug nearly always resulted in MDR clones. The resistance of these clones could be explained by reduced drug accumulation and was associated with a decrease rather than an increase in the low *MDR1* mRNA level. To test whether a decrease in *MDR1* mRNA indirectly affected resistance in these cells, we introduced a *MDR1*-specific hammerhead ribozyme into wild-type SW-1573 cells. Although this led to a substantial reduction in *MDR1* mRNA, it did not result in resistance. In all resistant clones we found an altered form of the multidrug resistance-associated protein (*MRP*), migrating slightly slower during SDS-polyacrylamide gel electrophoresis than *MRP* in parental cells. This altered MRP was also present in non-P-gp MDR somatic cell hybrids of the SW-1573 cells, demonstrating a clear linkage with the MDR phenotype. Treatment of crude cellular membrane fractions with *N*-glycanase, endoglycosidase H or neuraminidase showed that the altered migration of MRP on SDS-PAGE is due to a post-translational modification. There was no detectable difference in sialic acid content. In most but not all doxorubicin-selected clones, this MDR phenotype was accompanied by a reduction in topo IIα mRNA level. No reduction was found in the clones selected with vincristine. We conclude from these results that selection of the SW-1573 cell line for low levels of doxorubicin or vincristine resistance, predominantly results in MDR with reduced drug accumulation associated with the presence of an altered MRP protein. This mechanism can be accompanied by other resistance mechanisms, such as reduced topo IIα mRNA in case of doxorubicin selection.

**Keywords**: multidrug resistance; post-transcriptional modification; multidrug resistance-associated protein; reduced drug accumulation

Selection of the human non-small-cell lung cancer cell line SW-1573 for resistance to doxorubicin can result in two types of multidrug resistance (MDR): high-level resistance due to P-glycoprotein encoded by the *MDR1* gene (Baas *et al.*, 1990), a well-defined form of MDR (reviewed by Endicot and Ling, 1988; Roninson, 1991; Schinkel and Borst, 1991; Gottesman and Pastan, 1993; Moscow *et al.*, 1993); and low-level resistance associated with a complex phenotype that includes a decrease rather than an increase in *MDR1* expression, a decrease in topoisomerase (topo) IIα mRNA and a decreased drug uptake (Eijdems *et al.*, 1992). This form of non-P-gp MDR was first identified in three independent SW-1573 mutants (Keizer *et al.*, 1989; Baas *et al.*, 1990; Kuiper *et al.*, 1990) and it results in resistance to drugs affecting the function of topoisomerase II (topo II) such as daunorubicin, VP16-213 and *m*-AMSA [4′-(9-acridinylamino)methanesulphon-*m*-anisidine], to the membrane-active compound gramicidin D and to drugs affecting the polymerisation of microtubules such as the vinca alkaloids and colchicine (Keizer *et al.*, 1989; Baas *et al.*, 1990; Kuiper *et al.*, 1990; Eijdems *et al.*, 1992). In somatic cell fusions we have shown that the MDR phenotype, the reduced drug accumulation and the reduction in *MDR1* P-gp mRNA are transferred together to drug-sensitive SW-1573 cells, but that the alteration in topo IIα is not genetically linked to the non-P-gp MDR phenotype (Eijdems *et al.*, 1992).

Several other non-P-gp MDR cell lines have been selected (reviewed in Cole, 1992) and are being used to identify the mechanism(s) underlying this form of resistance. Cole *et al.* (1992), identified the multidrug resistance-associated protein (*MRP*) gene, which was amplified and overexpressed in one of these non-P-gp MDR cell lines, H69AR. Subsequently, overexpression of the *MRP* gene was reported in other non-P-gp MDR cell lines (Krishnamachary and Center, 1993; Slovak *et al.*, 1993; Zaman *et al.*, 1993; Barrand *et al.*, 1994; Schneider *et al.*, 1994), suggesting a role for MRP, a member of the ATP-binding cassette transporter superfamily, in these cell lines as well. Recent transfection studies have proved that MRP can confer resistance to a broad range of natural product drugs (Grant *et al.*, 1994; Zaman *et al.*, 1994) by extruding the drugs from the cells (Zaman *et al.*, 1994).

Since the multiplicity of alterations in the SW-1573 non-P-gp MDR cell lines described thus far remains puzzling, we have isolated a series of new resistant variants with drug concentrations just above the $IC_{10}$ of the parental cell line to study the following questions:

(1) What is the predominant form of drug resistance obtained by low-level drug selection?
(2) Are known transporters such as *MDR1* P-glycoprotein or MRP involved in the low-level MDR present in the resistant variants?
(3) Is down-regulation of *MDR1* mRNA an obligatory feature of low-level MDR in these variants?
(4) Is the observed resistance frequently associated with a decrease in topo IIα mRNA level?
(5) Does vincristine and doxorubicin selection result in the same resistance phenotype?

### Material and methods

### *Chemicals*

Doxorubicin (doxorubicin hydrochloride), vincristine sulphate, ouabain and digitonin (50% pure) were purchased from Sigma (St Louis, MO, USA), daunorubicin hydrochloride was obtained from Specia (Paris, France), [G-

Correspondence: P Borst
*Present address: Center for Molecular and Cellular Diagnostics, Cancer Center, University of New Mexico, Albuquerque, NM 87131-5636, USA
Received 22 November 1994; revised 16 March 1995; accepted 23 March 1995

$^3$H]vincristine sulphate (sp. act. 4.8 Ci mmol$^{-1}$) from Amersham (UK) and [G-$^3$H]daunorubicin (sp. act. 1.6 Ci mmol$^{-1}$) from DuPont de Nemours (Germany).

*Cell culture*

The resistant cell lines described in this study were derived from the human non-small-cell lung cancer cell line SW-1573, originally isolated and characterised by Dr A Leibovitz (Scott and White Clinic, Temple, TX, USA). Cell line S1*ou* is a subclone of the drug-sensitive parental cell line S1 (a SW-1573 subline which was morphologically homogeneous) transfected with the $\alpha_1$ subunit of the murine sodium/potassium exchanger (Eijdems *et al.*, 1992). This clone has similar growth characteristics and drug sensitivity as the original SW-1573 S1 cell line. The sensitivity for MDR drugs was also unaffected by the presence of ouabain in the culture medium. The non-P-gp MDR cell line 1R50b was isolated from the drug-sensitive parental cell line S1 by a multistep doxorubicin selection up to 50 nM (Keizer *et al.*, 1989; Baas *et al.*, 1990; Kuiper *et al.*, 1990), whereas cell line 5R30 was isolated by a single step at 30 nM doxorubicin as a single-cell clone. In this study, we decided to use a transfected single-cell subclone of S1 to exclude the possibility that we would select pre-existing MDR variants that might be present at a very low frequency in the original S1 cell line. All drug resistance assays and initial drug selection were performed in the absence of ouabain. After selection, MDR clones were tested for resistance to ouabain, to ascertain that the MDR clones were derived from S1*ou*. Resistant cell lines were isolated as single-cell clones from cell line S1*ou* in a single step at 25 nM, 30 nM and at 40 nM doxorubicin or at 20 nM and at 25 nM vincristine. For nomenclature we took the drug concentration (nM) used for selection followed by an unique clone number that is preceded by a 'V' when the clones were selected with vincristine. When the growth rate of the selected clones was similar to that of the parental cells without drug they were analysed. To generate stable transfectants containing the *MDR1*-specific hammerhead ribozyme, drug-sensitive S1 cells were transfected with the 196 *MDR1* ribozyme construct with linked neomycin resistance marker, designed by Kobayashi *et al.* (1993), following a standard calcium phosphate precipitation technique (Graham and Van der Eb, 1973). Control transfections were performed with pGEM3Zf(−) DNA. After 3 weeks of selection with G-418 (800 μg ml$^{-1}$), individual clones were picked and propagated separately under G-418 selection. All cells were grown as monolayers in Ham's F-10 medium (Gibco, Paisley, UK) supplemented with 10% fetal calf serum, 2 mM glutamine, penicillin (50 units ml$^{-1}$) and streptomycin (50 μg ml$^{-1}$). Cells were maintained in humidified air/5% carbon dioxide at 37°C. All cells were free of *Mycoplasma* as tested by the use of the Gen-Probe rapid *Mycoplasma* detection system (Gen-Probe, San Diego, CA, USA).

*Assay of drug resistance*

Clonogenic survival assays were carried out as follows: cells were plated at 80 cells per well in 24-well dishes (tissue culture cluster 3424; Costar, Cambridge, MA, USA) in the continuous presence of an increasing concentration of drugs. Cells were grown for 8 days, fixed and stained with 0.2% crystal violet (merck 820603) in 3.7% glutaraldehyde. The percentage of cells that were able to produce a colony of $>50$ cells was used as a measure of cell survival. The resistance was calculated as the ratio of $IC_{10}$ of the resistant cell line to the $IC_{10}$ of the parental cell line.

*Cellular drug accumulation*

Steady-state accumulation of drugs was measured according to Skovsgaard (1990), modified by Broxterman *et al.* (1988). Adherent cells in the logarithmic phase of growth were trypsinised, washed and resuspended at densities of $0.2-0.5 \times 10^6$ cells ml$^{-1}$ (daunorubicin) or $0.5-1.5 \times 10^6$ cells ml$^{-1}$ (vincristine) in Dulbecco's modified essential medium (DMEM) without bicarbonate containing 20 mM *N*-2-hydroxyethylpiperazine-*N*′-2ethanesulphonic acid (Hepes), 10% fetal calf serum (pH 7.35 ± 0.05) and divided into 0.5 ml portions. [G-$^3$H]vincristine (diluted with unlabelled vincristine to a vincristine concentration of 0.5 μM) or [G-$^3$H]daunorubicin (diluted with unlabelled daunorubicin to a daunorubicin concentration of 0.5 μM) was added and cells were incubated at 37°C for 60 min. In the same experiment the daunorubicin accumulation was compared with maximal daunorubicin binding to the cells upon permeabilisation of the plasma membrane with digitonin (Versantvoort *et al.*, 1992). Digitonin (20 μM) was added 5 min before the end of the incubation time with daunorubicin. Drug uptake was stopped by addition of ice-cold phosphate-buffered saline (PBS), supplemented with 10% growth medium, and after two cold washes the cells were transferred to liquid scintillation fluid Opti-Phase III (LKB, Bromma, Sweden) and radioactivity was measured. Values were corrected for the amount of cell-associated radioactivity at time zero at 0°C.

*RNAse protection assay*

Cytoplasmic RNA was isolated by a Nonidet P-40 lysis procedure (Sambrook *et al.*, 1989). The RNAse protection was carried out according to Zinn *et al.* (1983) modified by Baas *et al.* (1990). Ten micrograms of total cytoplasmic RNA from each SW-1573 derivative was used or 10 μg of *Escherichia coli* tRNA as a negative control (Boehringer Mannheim, Germany). The protected probe was visualised by electrophoresis through a denaturing 6% acrylamide gel containing 8 M urea, followed by autoradiography. The following probes were used: *MDR1*, a 301 nucleotide *MDR1* cDNA fragment (nucleotide positions 3500–3801; Chen *et al.*, 1986); topo IIα, a 174 nucleotide topo IIα cDNA fragment (nt positions 1343–1517; Tsai-Pflugfelder *et al.*, 1988); *MRP*, a 244 nucleotide *MRP* cDNA fragment (nucleotide positions 239–483; Zaman *et al.*, 1993). In all experiments a probe for γ-actin (Enoch *et al.*, 1986) was included as an internal control. To determine the intensity of the signals for *MDR1*, topo IIα, *MRP* and γ-actin mRNA bands, the autoradiographs were scanned on a Bioimage analysis system (Millipore, USA).

*Protein immunoblot analysis*

Total cell lysates were made by lysis of cells in 10 mM potassium chloride, 1.5 mM magnesium chloride, 10 mM Tris–HCl pH 7.4 and 0.5% (w/v) sodium dodecyl sulphate (SDS) supplemented with 1 mM phenylmethylsulphonyl fluoride (PMSF), leupeptide (2 μg ml$^{-1}$), pepstatin (1 μg ml$^{-1}$) and aprotinin (2 μg ml$^{-1}$). DNA was sheared by sonication. Crude cellular membrane fractions were prepared as described previously (Gerlach *et al.*, 1987) and protein concentrations were measured using the Bio-Rad protein assay (Bio-Rad, Richmond, CA, USA). The protein samples were dissolved in sample buffer [65 mM Tris–HCl pH 6.8, 2.5% (w/v) SDS, 5% (v/v) glycerol, 5% (v/v) 2-mercaptoethanol], separated on a 7.5% (w/v) polyacrylamide gel containing 0.1% (w/v) SDS and transferred onto nitrocellulose paper (Schleicher and Schuell, Dassel, Germany) by electroblotting. The blots were probed with the monoclonal anti-MRP antibody, MRPr1, raised against a bacterial fusion protein containing amino acids 192–360 of MRP (Flens *et al.*, 1994). Antibody was visualised with peroxidase-conjugated rabbit anti-rat immunoglobulins (Dako, Copenhagen, Denmark) followed by enhanced chemiluminescence detection (Amersham, UK).

*Analysis of protein-linked oligosaccharides*

For the removal of *N*-linked oligosaccharides, crude cellular membrane fractions were incubated with 2.67 mU μl$^{-1}$ *N*-glycanase (a mixture of endoglycosidase F and peptide-*N*-glycosidase F; Boehringer Mannheim, Mannheim, Germany)

overnight at 30°C as described previously (Schinkel *et al.*, 1993). High-mannose oligosaccharides were removed by incubation of the crude cellular membrane fractions with 2 units $ml^{-1}$ endoglycosidase H (Endo H; Boehringer Mannheim, Mannheim, Germany) in sodium citrate pH 5.5 (end concentration 50 mM) and 0.2% (w/v) SDS. Incubations were performed overnight at 37°C. For the removal of sialic acids, crude cellular membrane fractions were incubated with 10 units $ml^{-1}$ neuraminidase (type 8) overnight at 4°C.

### Results

### *MDR is frequently selected with low levels of doxorubicin*

SW-1573 clones, resistant to low levels of doxorubicin, were isolated in a single-step procedure using 25 nM, 30 nM and 40 nM doxorubicin ($IC_{10}$ of the parental cells is 15 nM). After 3 months of selection, 34 clones were isolated and cultured in the presence of doxorubicin. All clones were analysed for their level of mRNA for *MDR1* P-gp and topo IIα (see Figure 1) and a representative subset of ten clones was chosen and analysed in detail. As expected, all ten clones showed significant resistance to doxorubicin (Table I). With the exception of clone 5R30, all clones also showed some degree of vincristine resistance (Table I). We conclude that most clones have a MDR phenotype of the non-P-gp variety, as *MDR1* mRNA is reduced rather than elevated in most of them (see below).

### *Drug accumulation is reduced in the doxorubicin-selected SW-1573 clones*

To investigate whether drug resistance correlated with reduced drug accumulation in these cell lines, daunorubicin and vincristine accumulation was determined (Table II). All but two of the doxorubicin-selected clones (25.26 and 40.8) showed a reduced steady-state accumulation of daunorubicin compared with the drug-sensitive parental cell line (Table II). In most clones this accumulation defect for daunorubicin was due to a drug gradient over the plasma membrane because the drug uptake increased after permeabilisation of the cell membrane by digitonin (Table III). Despite the lack of an apparent daunorubicin accumulation defect, clone 40.8 showed a significant increase of drug accumulation in the presence of digitonin. No significant differences were found

**Table I** Drug resistance in the SW-1573 clones selected at a low doxorubicin or vincristine concentration

| Cell line | *Resistance factor (RF)*[a,b] *Doxorubicin* | *Vincristine* | *Doxorubicin RF/ vincristine RF* |
|---|---|---|---|
| *Doxorubicin-selected* | | | |
| 25.10 | 2.3 ± 0.7 (4)[c] | 1.7 ± 0.4 (4)[c] | 1.35 |
| 25.26 | 6.5 ± 1.3 (4)[c] | 3.2 ± 1.1 (5)[c] | 2.03 |
| 5R30 | 4.4 ± 0.1 (2)[c] | 1.1 ± 0.1 (2) | 4.00 |
| 30.3A | 2.4 ± 0.7 (3)[c] | 3.0 ± 2.0 (3) | 0.80 |
| 30.10 | 3.7 ± 1.4 (3)[c] | 2.4 ± 0.9 (3) | 1.54 |
| 30.12 | 3.2 ± 0.9 (3)[c] | 3.4 ± 1.4 (4)[c] | 0.94 |
| 30.14 | 4.3 ± 1.6 (4)[c] | 3.0 ± 1.2 (5)[c] | 1.43 |
| 40.3 | 4.8 ± 1.5 (3)[c] | 2.8 ± 0.9 (4)[c] | 1.71 |
| 40.8 | 4.2 ± 1.7 (4)[c] | 2.2 ± 0.8 (3) | 1.91 |
| 40.10 | 3.6 ± 1.5 (3)[c] | 2.8 ± 1.0 (4)[c] | 1.28 |
| 1R50b[d] | 6.1 ± 0.9 (5)[c] | 3.1 ± 0.8 (8)[c] | 1.96 |
| 30.3M | 3.7 ± 1.2 (3)[c] | 4.6 ± 1.0 (3)[c] | 0.80 |
| *Vincristine-selected* | | | |
| 20V1 | 3.1 ± 1.0 (3)[c] | 10.6 ± 1.0 (3)[c] | 0.29 |
| 20V2 | 3.0 ± 0.8 (3)[c] | 9.9 ± 2.1 (3)[c] | 0.30 |
| 20V5 | 2.9 ± 0.2 (3)[c] | 10.1 ± 2.5 (3)[c] | 0.29 |
| 25V3 | 2.9 ± 0.8 (3)[c] | 10.3 ± 1.3 (3)[c] | 0.28 |
| 25V4 | 2.9 ± 0.7 (3)[c] | 12.2 ± 1.8 (3)[c] | 0.24 |
| 25V5 | 2.9 ± 0.9 (3)[c] | 12.9 ± 1.1 (3)[c] | 0.22 |

[a]The resistance factor = $IC_{10}$ cell line/$IC_{10}$ parental cell line ($IC_{10}$ parental cell line S1 = $IC_{10}$ parental cell line S1*ou*). [b]The data are the mean resistance factor ± s.d. or the range in case of two experiments. The number of experiments (clonogenic survival assay) is in parentheses. Each experiment was performed in duplicate. [c]The resistance factor differs significantly from the resistance factor of the parental cell lines with at least 95% confidence. [d]Results for the original non-P-gp MDR cell line 1R50b are also shown.

**Figure 1** *MDR1* P-glycoprotein and topoisomerase IIα mRNA levels measured by RNAse protection assays. (**a**) *MDR1* mRNA levels. (**b**) Topoisomerase IIα mRNA levels. Clones are grouped by doxorubicin concentration used for selection. The identifying numbers of the clones are given on the *x*-axis. The mRNA levels are expressed relative to the mRNA level of the drug-sensitive parental cell line S1*ou*. The mRNA level of the parental cell line is 100% by definition. For the clones that were analysed in at least three independently isolated RNA preparations assayed in independent experiments, the mean mRNA (%) ± s.d. (error bar) is given. See also Table II for the ten clones analysed in detail.

**Table II** Topoisomerase IIα and *MDR1* mRNA levels of representative doxorubicin-selected SW-1573 cell lines and clone S1*rib* as determined by RNAse protection

| *Cell line* | *Topo II mRNA level (%)*[a] | MDR1 *mRNA level (%)*[a] |
|---|---|---|
| 25.10 | 70 ± 14 | 36 ± 13[b] |
| 25.26 | 58 ± 18[b] | 17 ± 3[b] |
| 5R30 | 46 ± 5[b] | 6 ± 1[b] |
| 30.3A | 13 ± 2[b] | 23 ± 11[b] |
| 30.10 | 56 ± 38 | 18 ± 14[b] |
| 30.12 | 108 ± 29 | 34 ± 20[b] |
| 30.14 | 49 ± 13[b] | 7 ± 1[b] |
| 40.3 | 29 ± 11[b] | 0.8 ± 0.8[b] |
| 40.8 | 123 ± 4 | 6 ± 2[b] |
| 40.10 | 45 ± 10[b] | 15 ± 11[b] |
| 1R50b | 13 ± 3[b] | 15 ± 6[b] |
| S1*rib* | ND | 13 ± 7[b] |

[a]The mRNA level of the parental cell line is 100% by definition. The data were obtained from three independently isolated RNA preparations assayed in three independent experiments and are presented as the mean mRNA (%) ± s.d. [b]The mRNA level is significantly reduced compared with the level of the parental cell line with at least 95% confidence.

between the $pH_i$ of the resistant and the parental cell lines (data not shown). This excludes a contribution of a $pH_i$ increase to the decreased accumulation of the weak base daunorubicin in the resistant cell lines. In addition, no differences in DNA content were found between any of the resistant clones and the parental cells (data not shown), excluding the possibility that the decrease in daunorubicin accumulation was due to a decreased amount of target DNA in the resistant cells.

Most resistant variants also accumulated less vincristine than the parental cells (Table III). This vincristine accumulation is corrected for differences in cell volume between resistant and parental cells as an increase in cell size might result in an increase in vincristine uptake and prevent the detection of an accumulation defect. A significant decrease in vincristine accumulation was detected for the clones isolated at the higher doxorubicin concentrations and a tendency towards decreased vincristine accumulation was found for the clones isolated at lower doxorubicin concentrations (25 nM/30 nM). Two clones, 5R30 and 25.26, showed no vincristine accumulation defect. Clone 5R30 was also not resistant to vincristine. Clone 25.26, however, is unusual in that it has a clear MDR phenotype despite its lack of a detectable accumulation defect for both daunorubicin or vincristine. There are two possibilities for the altered behaviour of clone 25.26. The first explanation is that this clone is multidrug resistant owing to a single mutation, and resistance in this clone is also due to a restricted access of drug to target. The alternative explanation would be that clone 25.26 carries two independent mechanisms for drug resistance, one conferring resistance to doxorubicin, like 5R30, and a second mutation conferring resistance to vincristine. This mutation, however, would have occurred in the absence of the selecting drug. In view of the very low frequency of spontaneously arising vincristine-resistant clones, we consider the scenario with two independent mutations highly unlikely. We assume that resistance in 25.26 is due to the restriction of drug to target, which somehow does not show up in our drug accumulation assays. Therefore, we have not included this clone in the statistical analysis regarding drug accumulation described below.

The results in Table II show that the resistance of the new clones is associated with the decreased drug accumulation in a qualitative sense. To analyse whether the level of drug accumulation also correlated with the degree of drug resistance in a quantitative sense, correlation coefficients were calculated for the parental cell line S1*ou* and its resistant derivatives. The unusual clone 25.26 was excluded from this analysis. A correlation coefficient of − 0.76 was obtained for vincristine accumulation and resistance, indicating that resistance is due to reduced drug accumulation ($P<0.01$). For the relation between reduced daunorubicin accumulation and resistance to the related anthracycline doxorubicin a low, non-significant, correlation coefficient of − 0.44 was found ($P>0.05$). This suggests that additional factors, such as alterations of topo IIα, affect doxorubicin resistance in these clones.

### MDR1 *P-gp mRNA levels are not increased in the SW-1573 clones with low-level MDR*

*MDR1* P-gp mRNA levels were measured in all 34 doxorubicin-selected clones by a RNAse protection assay (see Figure 1 for all clones and Table II for the ten clones analysed in detail). None of the resistant clones had elevated *MDR1* P-gp mRNA levels. On the contrary, nearly all clones showed some degree of *MDR1* mRNA reduction, whereas a decrease of *MDR1* mRNA levels of more than 50% was detectable in seven out of the ten clones isolated at 25 nM doxorubicin, in 14 out of the 16 clones selected at 30 nM and in all eight clones selected at 40 nM.

### *The reduction of* MDR1 *P-gp mRNA levels correlates with reduced vincristine accumulation*

All ten doxorubicin-selected clones analysed, showed a significant reduction of *MDR1* P-gp mRNA (Table II). To test whether a quantitative relation with drug resistance was present, correlation coefficients were calculated. The reduction of *MDR1* P-gp mRNA level and vincristine resistance showed a low, non-significant, correlation coefficient of − 0.47 ($P>0.05$). The reduction of *MDR1* P-gp mRNA level and decreased vincristine accumulation showed a non-significant correlation coefficient of 0.44 ($P>0.05$). However, omission of the data for clones 25.26 and 5R30, which do not show a vincristine accumulation defect, raises this last value to 0.91 ($P<0.01$).

### *Reduction of topoisomerase IIα gene expression is an additional event in the development of low-level doxorubicin resistance*

To determine the frequency of topo IIα mRNA reduction, all 34 doxorubicin-selected clones were analysed in RNAse protection assays. The topo IIα mRNA levels of the resistant clones varied from parental levels to less than 20% of parental levels (see Figure 1b and Table II for the ten clones analysed in detail). The decrease was at least 2-fold in 8 out of 16 clones isolated at 30 nM doxorubicin and in three out of seven clones isolated at 40 nM doxorubicin.

Statistical analysis of the data for the ten clones that were analysed in detail (Table II) showed a significant reduction of topo IIα levels in six clones (25.26, 5R30, 30.3, 30.14, 40.3, 40.10). This suggests that low doxorubicin concentrations can select for cells with lowered topo IIα mRNA levels.

FACS analysis showed no significant differences in cell cycle distribution between the clones and the parental cell lines that could account for the observed topo IIα mRNA reduction and the variation between the clones (data not shown). The alterations in topo IIα mRNA level were not due to changes in γ-actin levels, since similar results were obtained with a probe for β-glucuronidase as internal standard in the RNAse protection experiments (data not shown). The only exception was clone 40.8, which had an increased cell size and increased ratio of γ-actin to β-glucuronidase mRNA in the absence of a decreased topo IIα mRNA level.

### *MDR clones selected at low levels of vincristine do not exhibit the decrease in topoisomerase IIα mRNA*

To test whether a similar type of non-P-gp MDR could be obtained with vincristine as with doxorubicin, we selected an additional set of clones in a single step with 20 or 25 nM

**Table III** Daunorubicin and vincristine accumulation in SW-1573 clones selected at low doxorubicin or vincristine concentration

| | *Daunorubicin accumulation*[a] | | | | |
|---|---|---|---|---|---|
| | *Control* | | *Digitonin* | *Vincristine accumulation*[a] | |
| *Cell line* | *pmol* $10^{-6}$ *cells* | *%* | *pmol* $10^{-6}$ *cells* | *pmol* $10^{-6}$ *cells* | *%* |
| *Doxorubicin-selected* | | | | | |
| S1*ou* | 215 ± 11 | 100 | 207 ± 30 | 21.1 ± 6.7 | 100 |
| 1R50b | 146 ± 27[c] | 68 | 198 ± 41[c] | 11.1 ± 3.9[c] | 52 |
| 25.10 | 180 ± 31[c] | 84 | 239 ± 19[c] | 14.7 ± 4.8 | 70 |
| 25.26 | 196 ± 22 | 91 | 196 ± 40 | 26.5 ± 5.9 | 125 |
| 5R30 | 156 ± 25[c] | 72 | 215 ± 42[c] | 19.8 ± 0.5 | 94 |
| 30.3A | 130 ± 23[c] | 60 | 221 ± 51[c] | 11.6 ± 3.7[c] | 55 |
| 30.10 | 156 ± 15[c] | 72 | 258 ± 16[c] | 12.3 ± 6.4 | 58 |
| 30.12 | 165 ± 19[c] | 77 | 208 ± 16[c] | 13.8 ± 4.1 | 65 |
| 30.14 | 182 ± 26[c] | 85 | 208 ± 50 | 9.1 ± 2.6[c] | 43 |
| 40.3 | 142 ± 25[c] | 66 | 195 ± 34[c] | 4.13 ± 2.0[c] | 20 |
| 40.8 | 202 ± 29 | 94 | 250 ± 32[c] | 11.4 ± 3.7[c] | 54 |
| 40.10 | 172 ± 29[c] | 80 | 230 ± 21[c] | 10.3 ± 3.3[c] | 49 |
| *Vincristine-selected* | | | | | |
| S1*ou*[d] | 331 ± 12 | 100 | 290 ± 43 | 27.9 ± 8.6 | 100 |
| 20V2 | 175 ± 29[c] | 53 | 284 ± 36[c] | 7.4 ± 1.7[c] | 30 |
| 25V4 | 154 ± 13[c] | 47 | 296 ± 21[c] | 7.2 ± 1.7[c] | 28 |

[a]The data are from 3–8 independent experiments each performed in quadruplicate. The mean accumulation ± s.d. is given. [b]The vincristine accumulation is corrected for differences in cell volume compared with the parental cell line as measured by a Coulter counter calibrated with microbeads. The standard deviation is obtained from the standard deviations of both the vincristine accumulation and the cell volume. [c]The accumulation differs significantly from that in the S1*ou* cells or the accumulation after addition of digitonin differs significantly from that without addition of digitonin ($P < 0.05$) according to the unpaired Student's *t*-test. [d]As the drug accumulation data for the doxorubicin- and for the vincristine-selected cell lines were determined in independent experiments, the drug accumulation of the drug-sensitive parental cell line for both data sets is given.

vincristine ($IC_{10}$ of the parental cell line is 2.5 nM). These were the lowest concentrations that still fully eliminated parental background clones. After 6 months of selection, six clones were isolated and cultured in the presence of vincristine. The analysis of these clones is summarised in Figure 2 and Tables I and IV. The vincristine-selected clones resemble their doxorubicin-selected counterparts in several characteristics, i.e. resistance to both doxorubicin and vincristine (Table I), decreased drug accumulation (Table III) and a reduced *MDR1* P-gp mRNA level (Figure 2). However, they differ from the doxorubicin-selected clones in two properties: they lack the reduced topo IIα mRNA level (Figure 2) and they have a much higher vincristine than doxorubicin resistance (Table I). Whereas the average ratio doxorubicin/vincristine resistance was 1.6 (range 0.8–4.0) for the ten doxorubicin-selected clones, it was only 0.3 (range 0.2–0.3) for the six vincristine-selected ones (Table I).

### *The reduction of* MDR1 *P-gp mRNA does not contribute to drug resistance*

We showed that the reduction of *MDR1* P-gp mRNA was co-transferred with the MDR phenotype in somatic cell fusion experiments (Eijdems *et al.*, 1992) and was present in all MDR SW-1573 cells in this study (Figures 1 and 2, Table II). The *MDR1* P-gp extrudes drugs from cells and one would therfore not expect a decrease in *MDR1* expression to increase resistance. To exclude exotic indirect effects, we artificially reduced the *MDR1* in the parental drug-sensitive cells by transfection of a hammerhead ribozyme specific for *MDR1* (Kobayashi *et al.*, 1993, 1994). Transfected cells were selected for G-418 resistance, as a neomycin phosphotransferase gene was present in the expression vector. One of ten G-418-resistant subclones, clone S1*rib*, showed a reduced *MDR1* mRNA level similar to that of the SW-1573 non-P-gp MDR cells (Table II), but no detectable decrease or increase of sensitivity to doxorubicin, vincristine or VP16-213 was found for this clone in clonogenic survival assays (data not shown). These results indicate that, despite the observed down-regulation of *MDR1* mRNA in the MDR cell lines, reduced *MDR1* mRNA levels do not contribute to drug resistance in the SW-1573 cells.

**Figure 2** RNAse protection assays to quantify *MDR1* P-gp, *MRP* and topoisomerase IIα mRNA levels in the SW-1573 clones selected with vincristine. The vincristine concentration used for selection (nM) is used for nomenclature, followed by an identifying clone number. For comparison, RNA of the original non-P-gp MDR cell line 1R50b was also assayed. Ten micrograms of total cytoplasmic RNA from each SW-1573 derivative was used. The γ-actin signal was comparable for the independent experiments. On a longer exposure a very faint band of *MDR1* mRNA signal was detectable in the vincristine-selected clones and was similar to the signal in the 1R50b cell line.

### MRP *mRNA levels in the resistant SW-1573 clones*

All new MDR clones showed a slight increase in *MRP* mRNA level (Figures 2 and 3, Table IV). For most individual clones this small increase was not statistically significant, as also observed in initial experiments with the resistant cell line 1R50b (Zaman *et al.*, 1993). We observed large variations in independently isolated RNA preparations, illustrated by the standard deviations in Table IV, e.g. 1R50b 129% ± 79. Only two individual clones, 30.12 and 30.3M, showed a significant increase in *MRP* mRNA (with at least 95% confidence; Table IV and Figure 3). In 30.3M, obtained by continued doxorubicin selection of 30.3A, this increase was due to transcriptional activation of the *MRP* gene (Eijdems *et al.*, 1995*a*), which was accompanied by an increase in both doxorubicin and vincristine resistance compared with 30.3A (Table I). This suggests that *MRP* can be involved in low-level drug resistance in the SW-1573 cell lines. Despite large variability in *MRP* mRNA levels in the individual clones, all clones taken as a group had a significant increase of MRP mRNA level relative to the parental cells (*t*-test, $P<0.01$).

### *An altered form of MRP in the MDR SW-1573 cells*

To test whether the slight increase in *MRP* mRNA found in the resistant variants was translated into an increased level of MRP protein, we analysed the level of MRP in these variants by immunoblotting. Cell extracts were size fractionated by SDS-PAGE and MRP was detected on blots with a monoclonal antibody raised against a MRP fusion protein (Flens *et al.*, 1994). Figure 4a and b shows no major increase in MRP levels in these clones, in line with the mRNA results. However, the resistant clones contained MRP with an aberrant migration pattern in SDS-PAGE. A band with an

**Table IV** *MRP* mRNA levels of low-resistant MDR SW-1573 cell lines selected with doxorubicin or vincristine

| *Cell line* | MRP *mRNA level (%)*[a] |
|---|---|
| *Doxorubicin-selected* | |
| 25.10 | 161 ± 102 |
| 30.12 | 218 ± 82[b] |
| 40.3 | 171 ± 57 |
| 1R50b | 129 ± 79 |
| 30.3M | 292 ± 64[b] |
| *Vincristine-selected* | |
| 20V2 | 198 ± 86 |
| 25V4 | 189 ± 73 |

[a]The *MRP* mRNA of the parental cell line is 100% by definition. The data were obtained from three independently isolated RNA preparations assayed in three independent experiments and are presented as the mean mRNA (%) ± s.d. [b]The mRNA level differs significantly from that of the parental cell line with at least 95% confidence.

**Figure 3** RNAse protection assay to quantify *MRP* mRNA levels in the SW-1573 clones selected with doxorubicin. The doxorubicin concentration used for selection (nM) is used for the nomenclature, followed by an identifying clone number. For comparison, RNA of the original non-P-gp MDR cell line 1R50b was also assayed. Ten micrograms of total cytoplasmic RNA from each SW-1573 derivative was used. The positions of the protected fragments of *MRP* and γ-actin mRNA as well as the size (nucleotides) of the molecular weight markers are indicated (M).

**Figure 4** Western blot analysis of MRP in SW-1573 cells. (**a** and **b**) MRP in total cell lysates of drug-sensitive cell line S1*ou* and low-resistance SW-1573 cell lines. The cell lines were selected with doxorubicin (25.10, 30.3A, 30.3M, 30.12, 40.3 and 1R50b) or vincristine (20V2 and 25V4), or were derived by somatic cell fusion between cell line 1R50b and drug-sensitive, parental cell line S1*ou* (F6.1 and F6.3). (**c**) Crude cellular membrane fractions of S1*ou*, 1R50b, 30.3M and 20V2, incubated in the absence (−) or presence (+) of *N*-glycanase. The protein samples (30 μg of protein per lane for **a** and **b**, and 20 μg for **c**) were size fractionated in a 7.5% polyacrylamide gel containing 0.5% SDS, transferred to a nitrocellulose membrane and MRP was detected by incubation with monoclonal antibody MRPr1. The size (kDa) and position of molecular weight markers are indicated.

apparent molecular mass of 190–200 kDa was present, whereas parental MRP runs at 180 kDa (Figure 4a and b). The relative amounts of the 180 kDa and the 190–200 kDa forms of MRP varied somewhat in different clones. The increase in resistance level in cell line 30.3M compared with 30.3A (Table I) was accompanied by a nearly complete replacement of the 180 kDa protein by the 190 –200 kDa protein (Figure 4a). The protein with altered mobility was also detectable in the non-P-gp MDR cell hybrids F6.1 and F6.3 (Figure 4b), which were derived by somatic cell fusion of the non-P-gp MDR cell line 1R50b with drug-sensitive cell line S1*ou* (Eijdems *et al.*, 1992). This shows that the presence of the 190–200 kDa MRP protein is linked to the non-P-gp MDR phenotype in the SW-1573-derived cell lines.

To analyse the nature of the altered behaviour of MRP on SDS-PAGE, crude cellular membrane fractions of the multidrug-resistant cell lines 1R50b, 30.3M (doxorubicin selected) and 20V2 (vincristine selected) were analysed (Figure 4c). In the membrane fractions, a single MRP band with a molecular weight in the range of 190–200 kDa was detectable in the resistant cells, in contrast to the 180 kDa protein detectable in the parental, drug-sensitive cell line S1*ou*. Similar to the results obtained with total cell lysates, no major increase in the amount of MRP and protein was detectable in the resistant cell lines (1R50b, 20V2). Only cell line 30.3M, which has a 3-fold elevated MRP mRNA, also showed an increase of MRP epitopes in the membrane fractions.

Treatment of the crude cellular membrane fractions with *N*-glycanase, which removes *N*-linked oligosaccharides, resulted in the appearance of a single band in the range of 150 kDa in both the parental and in the resistant cell lines (Figure 4c). This indicates that the altered mobility of MRP in the resistant cell lines is due to differences in post-translational modification and not to major differences in the protein backbone.

Treatment of purified membrane fractions with endoglycosidase H (Endo H), which removes high-mannose oligosaccharides, did not affect the mobility of MRP either in the resistant cell lines or in the drug-sensitive cell line S1*ou* (data not shown). Removal of sialic acid with neuraminidase reduced the size of MRP protein in both the drug-resistant and the drug-sensitive cells to the same extent and did not alter the difference in mobility (data not shown). These results demonstrate that the 180 kDa and the 190–200 kDa MRP proteins both contain complex oligosaccharides and must have passed the medial Golgi compartment and reached the trans-Golgi network.

### Discussion

We have analysed a large set of drug-resistant variants of the human lung cancer line SW-1573, selected with low concentrations of either doxorubicin or vincristine. We found that the predominant type of resistance is a form of MDR characterised by reduced drug accumulation and decreased *MDR1* P-gp mRNA levels. Since the degree of resistance correlates with the extent to which drug accumulation is diminished, it seems likely that the lowered intracellular drug concentrations are mainly responsible for resistance in these variants. A diminished drug accumulation has also been observed in other non-P-gp MDR cell lines (reviewed by Cole, 1992), the H69/AR cell line being the only exception (Cole *et al.*, 1991).

Overexpression of the *MRP* gene can confer multidrug resistance associated with reduced drug accumulation in human HeLa cells (Grant *et al.*, 1994) and SW-1573 cells (Zaman *et al.*, 1994). In several non-P-gp MDR cell lines a role for overexpression of *MRP* was suggested as well (Krishnamachary and Center, 1993; Slovak *et al.*, 1993; Zaman *et al.*, 1993; Barrand *et al.*, 1994; Schneider *et al.*, 1994). The *MRP* mRNA and MRP protein levels in most clones isolated in this study were increased at most 2-fold. At first sight, it seems unlikely that this could account for the complete MDR phenotype in these SW-1573 cells. However, our finding that an altered form of MRP is present in all our non-P-gp MDR clones and that this altered form co-segregates with the non-P-gp MDR trait in somatic cell fusion experiments strongly indicates that MRP is involved in the non-P-gp phenotype of these clones. To test this more directly, we are constructing a ribozyme directed against MRP mRNA.

The precise nature of the alteration of MRP resulting in altered migration in SDS-PAGE gels is not yet known. Our results show that this altered mobility is due to a change in the MRP-linked complex oligosaccharides. Post-translational modification is the most plausible explanation. This modification could either be directly responsible for MDR, e.g. result in a more active MRP drug pump, or it could be a consequence of other alterations affecting MRP, such as altered routing in the Golgi apparatus. Minor changes in the primary structure of MRP cannot be excluded yet.

A remarkable feature of the non-P-gp MDR SW-1573 clones is the paradoxical decrease of *MDR1* mRNA levels. This was also reported for the H69-AR cell line (Cole *et al.*, 1991), the non-P-gp MDR cell line from which the *MRP* gene was isolated (Cole *et al.*, 1992). Although it is hard to believe that a decrease in *MDR1* P-glycoprotein could result in an increased drug extrusion, the theoretical possibility existed that P-glycoprotein was extruding an endogenous compound required to induce the non-P-gp MDR mechanism in SW-1573 cells. Our ribozyme experiment excludes this far-fetched possibility. Hence, the down-regulation of *MDR1* does not cause MDR in our resistant clones, but is a side-effect of the mutation causing the non-P-gp phenotype. In view of the alteration of MRP in the SW-1573 cells, the decrease in *MDR1* mRNA could be an indirect effect of the post-translational modification of a regulator of *MDR1* expression. The absence of increased *MDR1* expression in the non-P-gp MDR cell is not due to the inability of the SW-1573 cells to activate the *MDR1* gene since we have shown that SW-1573 cells selected for higher levels of resistance invariably increase their expression of *MDR1* (Baas *et al.*, 1990).

The non-P-gp MDR phenotype with altered MRP, reduced drug uptake and reduced *MDR1* mRNA was obtained both with doxorubicin and vincristine selection. However, the resistance spectra of the two types of clones differed (Table I). This suggests that cells may activate multiple resistance mechanisms, even at these low levels of selection. In the case of doxorubicin selection, the supplementary resistance mechanism probably consists of alterations in topo IIα. Most of the MDR variants selected for doxorubicin resistance contained decreased topo IIα mRNA levels, and the clones with normal mRNA level may nevertheless contain decreased topo II enzyme activity (Eijdems *et al.*, 1995*b*). In the case of vincristine selection, we have no clue as to the mechanism that contributes to the relatively high vincristine resistance.

Our experiments show that low-level resistance associated with altered MRP arises with high frequency in SW-1573 cells. Precise estimates of this frequency are not available, as the sensitive cells continue to divide slowly during mutant selection at low drug concentration. It is clearly much higher, however, than the activation of *MDR1*, which occurs at a frequency of $1-2 \times 10^{-6}$ per cell in SW-1573 when higher drug concentrations are used for selection (Eijdems *et al.*, 1992, and unpublished results). We infer from our results that resistance associated with altered MRP only protects against relatively low levels of resistance. This follows from the association of this type of resistance with other forms of low-level resistance, topo II alterations or unknown, and from the fact that selection for higher levels of resistance results either in an activation of *MDR1* or in a transcriptional activation of *MRP*. The only clear example of *MRP* activation is clone 30.3M, which has a 3-fold increase in *MRP* mRNA. We have recently shown by RNA run-on experiments that this is due to increased transcription of *MRP* (Eijdems *et al.*, 1995*b*).

In conclusion, we have shown that in the SW-1573 lung cancer cell line low levels of two different types of drug select for altered MRP accompanied by reduced drug accumulation and concomitant multidrug resistance. Although this MDR phenotype correlated well with reduced *MDR1* mRNA, we excluded a contribution of this reduction to drug resistance. Our results also show that, even at the low drug concentrations used, multiple resistance mechanisms may coexist in resistant clones.

**Acknowledgements**
We thank Dr AH Schinkel for critical reading of the manuscript, Dr HJ Broxterman, Dr J Lankelma and S Mülder for helpful discussions during this study and for helping with and allowing us to perform some of the experiments presented in this paper, Dr H Kobayashi for providing the *MDR1* ribozyme construct, Dr J Schornagel for help during the analysis of the clones transfected with the *MDR1* ribozyme construct and E Nooteboom for FACSscan analysis. This study was supported by the Dutch Cancer Society Grant NKI 91-18 (to FB and PB).

**References**

BAAS F, JONGSMA APM, BROXTERMAN HJ, ARCECI RJ, HOUSMAN D, SCHEFFER GL, RIETHORST A, VAN GROENIGEN M, NIEUWINT AWM AND JOENJE H. (1990). Non-P-glycoprotein-mediated mechanism for multidrug resistance precedes P-glycoprotein expression during *in vitro* selection for doxorubicin resistance in a human lung cancer cell line. *Cancer Res.*, **50**, 5392–5398.

BARRAND MA, HEPPEL-PARTON AC, WRIGHT KA, RABBITTS PH AND TWENTYMAN PR. (1994). A 190-kilodalton protein overexpressed in non-P-glycoprotein-containing multidrug-resistant cells and its relationship to the MRP gene. *J. Natl Cancer Inst.*, **86**, 110–117.

BROXTERMAN HJ, KUIPER CM, SCHUURHUIS GJ, TSURUO T, PINEDO HM AND LANKELMA J. (1988). Increase of daunorubicin and vincristine accumulation in multidrug resistant human ovarian carcinoma cells by a monoclonal antibody reacting with P-glycoprotein. *Biochem. Pharmacol.*, **37**, 2389–2393.

CHEN C, CHIN JE, UEDA K, CLARK DP, GOTTESMAN MM AND RONINSON IB. (1986). Internal duplication and homology with bacterial transport proteins in the *mdr*1 (P-glycoprotein) gene from multidrug-resistant human cells. *Cell*, **47**, 381–389.

COLE SPC. (1992). The 1991 Merck Frosst Award. Multidrug resistance in small cell lung cancer. *Can. J. Physiol. Pharmacol.*, **70**, 313–329.

COLE SPC, CHANDA ER, DICKE FP, GERLACH JH AND MIRSKI SEL. (1991). Non-P-glycoprotein-mediated multidrug resistance in a small cell lung cancer cell line: evidence for decreased susceptibility to drug-induced DNA damage and reduced levels of topoisomerase II. *Cancer Res.*, **51**, 3345–3352.

COLE SCP, BHARDWAJ G, GERLACH JH, MACKIE JE, GRANT CE, ALMQUIST KC, STEWART AJ, KURZ EU, DUNCAN AMV AND DEELEY RG. (1992). Overexpression of a transporter gene in a multidrug-resistant human lung cancer cell line. *Science*, **258**, 1650–1654.

EIJDEMS EWHM, BORST P, JONGSMA APM, DE JONG S, DE VRIES EGE, VAN GROENIGEN M, VERSANTVOORT CHM, NIEUWINT AWM AND BAAS F. (1992). Genetic transfer of non-P-glycoprotein-mediated multidrug resistance (MDR) in somatic cell fusion: dissection of a compound MDR phenotype. *Proc. Natl Acad. Sci. USA*, **89**, 3498–3502.

EIJDEMS EHWM, DE HAAS M, COCO-MARTIN JM, OTTENHEIM CPE, ZAMAN GJR, DAWERSE JG, BRUENING MH, TWENTYMAN PR, BORST P AND BAAS F. (1995*a*). Mechanisms of *MRP* overexpression in four human lung cancer cell lines and analysis of the *MRP* amplicon. *Int. J. Cancer*, **60**, 676–684.

EIJDEMS EWHM, DE HAAS M, TIMMERMAN AJ, VAN DER SCHANS GP, DE NOOY J, ASTALDI RICOTTI GCB, BORST P AND BAAS F. (1995*b*). Reduced topoisomerase II activity in multidrug resistant human non-small-cell lung cancer cell lines. *Br. J. Cancer*, **71**, 40–47.

ENDICOTT JA AND LING V. (1988). The biochemistry of P-glycoprotein-mediated multidrug resistance. *Annu. Rev. Biochem.*, **58**, 137–171.

ENOCH T, ZINN K AND MANIATIS T. (1986). Activation of the human β-interferon gene requires an interferon inducible factor. *Mol. Cell. Biol.*, **6**, 801–810.

FLENS MJ, IZQUIERDO MA, SCHEFFER GL, FRITZ JM, MEIJER CJLM, SCHEPER RJ AND ZAMAN GJR. (1994). Immunochemical detection of the multidrug resistance-associated protein MRP in human multidrug-resistant tumor cells by monoclonal antibodies. *Cancer Res.*, **54**, 4557–4563.

GERLACH JH, BELL DR, KARAKOUSIS C, SLOCUM HK, KARTNER N, RUSTUM YM, LING V AND BAKER RM. (1987). P-glycoprotein in human sarcoma: evidence for multidrug resistance. *J. Clin. Oncol.*, **5**, 1452–1460.

GOTTESMAN MM AND PASTAN I. (1993). Biochemistry of multidrug resistance mediated by the multidrug transporter. *Annu. Rev. Biochem.*, **62**, 385–427.

GRAHAM FL AND VAN DER EB AJ. (1973). A new technique for the assay of infectivity of human adenovirus 5 DNA. *Virology*, **52**, 456–467.

GRANT CE, VALDIMARSSON G, HIPFNER DR, ALMQUIST KC, COLE SPC AND DEELEY RG. (1994). Overexpression of multidrug resistance-associated protein (MRP) increases resistance to natural product drugs. *Cancer Res.*, **54**, 357–361.

KEIZER HG, SCHUURHUIS GJ, BROXTERMAN HJ, LANKELMA J, SCHOONEN W AND JOENJE H. (1989). Correlation of multidrug resistance with decreased drug accumulation, altered subcellular drug distribution and increased P-glycoprotein expression in cultured SW-1573 human lung tumor cells. *Cancer Res.*, **49**, 2988–2993.

KOBAYASHI H, DORAI T, HOLLAND JF AND OHNUMA T. (1993). Cleavage of human *MDR1* mRNA by a hammerhead ribozyme. *FEBS Lett.*, **319**, 71–74.

KOBAYASHI H, DORAI T, HOLLAND JF AND OHNUMA T. (1994). Reversal of drug sensitivity in multidrug-resistant tumor cells by an *MDR1* (*PGY*1) ribozyme. *Cancer Res.*, **54**, 1271–1275.

KRISHNAMACHARY N AND CENTER MS. (1993). The *MRP* gene associated with a non-P-glycoprotein multidrug resistance encodes a 190-kDa membrane bound glycoprotein. *Cancer Res.*, **53**, 3658–3661.

KUIPER CM, BROXTERMAN HJ, BAAS F, SCHUURHUIS GJ, HAISMA HJ, SCHEFFER GL, LANKELMA J AND PINEDO HM. (1990). Drug transport variants without P-glycoprotein overexpression from a human squamous lung cancer cell line after selection with doxorubicin. *J. Cell. Pharmacol.*, **1**, 35–41.

MOSCOW JA, SCHNEIDER E AND COWAN KH. (1993). Multidrug resistance. *Cancer Chemother. Biol. Res. Modif.*, **14**, 98–117.

RONINSON IB. (1991). *Molecular and Cellular Biology of Multidrug Resistance in Tumor Cells*. Plenum: New York.

SAMBROOK J, FRITSCH EF AND MANIATIS T. (1989). *Molecular Cloning: A Laboratory Manual*, 2nd edn. Cold Spring Harbor Laboratory Press: Cold Spring Harbor, NY.

SCHINKEL AH AND BORST P. (1991). Multidrug resistance mediated by P-glycoproteins. *Semin. Cancer Biol.*, **2**, 213–226.

SCHINKEL AH, KEMP S, DOLLÉ M, RUDENKO G AND WAGENAAR E. (1993). *N*-glycosylation and deletion mutants of the human *MDR1* P-glycoprotein. *J. Biol. Chem.*, **268**, 7474–7481.

SCHNEIDER E, HORTON JK, YANG C-H, NAKAGAWA M AND COWAN KH. (1994). Multidrug resistance-associated protein gene overexpression and reduced drug sensitivity of topoisomerase II in a human breast carcinoma MCF7 cell line selected for etoposide resistance. *Cancer Res.*, **54**, 152–158.

SKOVSGAARD T. (1990). Transport and multi-drug resistance. Identifications of a changed pH gradient across the membrane. In *Pezcoller Foundation Symposia, Drug Resistance: Mechanisms and Reversal*, Vol. 1, Mihich E (ed.) pp. 209–231. John Libbey CIC: Rome.

SLOVAK ML, PELKEY HO J, BHARDWAY G, KURZ EU, DEELEY RG AND COLE SPC. (1993). Localization of a novel multidrug resistance-associated gene in the HT1080/DR4 and H69AR human tumor cell lines. *Cancer Res.*, **53**, 3221–3225.

TSAI-PFLUGFELDER M, LUI LF, LIU AA, TEWEY KM, WHANG-PENG J, KNUTSEN T, HUEBNER K, CROCE CM AND WANG JC. (1988). Cloning and sequencing of cDNA encoding human DNA topoisomerase II and localization of the gene to chromosome region 17q21–22. *Proc. Natl Acad. Sci. USA*, **85**, 7177–7181.

VERSANTVOORT CHM, BROXTERMAN HJ, FELLER N, DEKKER H, KUIPER CM AND LANKELMA J. (1992). Probing daunorubicin accumulation defects in non-P-glycoprotein expressing multidrug-resistant cell lines using digitonin. *Int. J. Cancer*, **50,** 906–911.

ZAMAN GJR, VERSANTVOORT CHM, SMIT JJM, EIJDEMS EWHM, DE HAAS M, SMITH AJ, BROXTERMAN HJ, MÜLDER NH, DE VRIES EGE, BAAS F AND BORST P. (1993). Analysis of the expression of *MRP*, the gene for a new putative transmembrane drug transporter, in human multidrug resistant lung cancer cell lines. *Cancer Res.*, **53,** 1747–1750.

ZAMAN GJR, FLENS MJ, VAN LEUSDEN MR, DE HAAS M, SCHEPER RJ, MÜLDER HS, LANKELMA J, PINEDO HM, BAAS F, BROXTERMAN HJ AND BORST P. (1994). The human multidrug resistance-associated protein (*MRP*) is a plasma membrane drug efflux pump. *Proc. Natl Acad. Sci. USA*, **91,** 8822–8826.

ZINN K, DIMAIO D AND MANIATIS T. (1983). Identification of two distinct regulatory regions adjacent to the human β-interferon gene. *Cell*, **34,** 865–879.